# Bone marrow mesenchymal stem cell-derived exosomal miR-21a-5p alleviates renal fibrosis by attenuating glycolysis by targeting PFKM

**DOI:** 10.1038/s41419-022-05305-7

**Published:** 2022-10-17

**Authors:** Shihao Xu, Yin Celeste Cheuk, Yichen Jia, Tian Chen, Juntao Chen, Yongsheng Luo, Yirui Cao, Jingjing Guo, Lijun Dong, Yi Zhang, Yi Shi, Ruiming Rong

**Affiliations:** 1 Department of Urology, Zhongshan Hospital, Fudan University, Shanghai, 200032 China; 2 Shanghai Key Laboratory of Organ Transplantation, Shanghai, 200032 China; 3 Department of Urology, Huashan Hospital, Fudan University, Shanghai, 200040 China; 4 Operation Room, Shanghai Tenth People’s Hospital, Tongji University, Shanghai, 200072 China; 5 Institute of Clinical Science, Zhongshan Hospital, Fudan University, Shanghai, 200032 China; 6 Department of Transfusion, Zhongshan Hospital, Fudan University, Shanghai, 200032 China

**Keywords:** Mesenchymal stem cells, Chronic kidney disease

## Abstract

Renal fibrosis is a common pathological feature and outcome of almost all chronic kidney diseases, and it is characterized by metabolic reprogramming toward aerobic glycolysis. Mesenchymal stem cell-derived exosomes (MSC-Exos) have been proposed as a promising therapeutic approach for renal fibrosis. In this study, we investigated the effect of MSC-Exos on glycolysis and the underlying mechanisms. We demonstrated that MSC-Exos significantly ameliorated unilateral ureter obstruction (UUO)-induced renal fibrosis by inhibiting glycolysis in tubular epithelial cells (TECs). miRNA sequencing showed that miR-21a-5p was highly enriched in MSC-Exos. Mechanistically, miR-21a-5p repressed the expression of phosphofructokinase muscle isoform (PFKM), a rate-limiting enzyme of glycolysis, thereby attenuating glycolysis in TECs. Additionally, knockdown of miR-21a-5p abolished the renoprotective effect of MSC-Exos. These findings revealed a novel role for MSC-Exos in the suppression of glycolysis, providing a new insight into the treatment of renal fibrosis.

## Background

Chronic kidney disease (CKD) has emerged as a severe public health problem, with a global prevalence of 8–16% [1]. Regardless of its etiology, renal fibrosis tends to be the eventual pathological outcome of CKD and is characterized by excessive extracellular matrix deposition [2, 3]. However, effective means to ameliorate or even reverse renal fibrosis remain scarce. Therefore, it is of great clinical significance to develop a treatment for renal fibrosis.

The kidney is one of the most metabolically active organs and requires a large amount of energy to maintain its normal physiological structure and function. When supplied with adequate oxygen, the majority of cells generate energy through the aerobic oxidation of glucose. Only in the absence of oxygen, cells produce energy and large amounts of lactate through anaerobic glycolysis. Conversely, most tumor cells rely primarily on glycolysis for energy even in the presence of adequate oxygen, which is referred to as “aerobic glycolysis” or the “Warburg effect” [4, 5]. Although aerobic glycolysis was originally discovered in tumor cells, it is now increasingly recognized as an important pathogenic process in renal fibrosis. More importantly, abnormalities in glucose metabolism promote the progression of renal fibrosis [6, 7]. Therefore, altering the pattern of metabolism may be a novel strategy to prevent renal fibrosis.

Mesenchymal stem cells (MSCs) have been developed as a prospective treatment option for CKD due to their trophic, anti-inflammatory and immunomodulatory effects [8–10]. However, there are numerous challenges for their clinical application, including safety, tumorigenesis and immunosuppression [11]. Exosomes are a type of extracellular vesicles (EVs) with a size range of 50 to 150 nm in diameter that are secreted by almost all kinds of cells, [12] and they shuttle a variety of molecules, including proteins, lipids, DNA, mRNA and microRNAs (miRNAs), from parental cells to other cells to regulate intercellular communication [13, 14]. MSC-Exos are endowed with several advantages over MSCs, such as higher safety, lower immunogenicity and avoidance of tumor formation [15]. Thus, MSC-Exos have the potential to be an alternative to MSC-based therapy. Recently, studies have reported that MSC-Exos inhibit glycolysis in certain diseases. For instance, MSC-Exos inhibit glycolysis in pulmonary artery smooth muscle cells and promote oxidative phosphorylation in a model of pulmonary arterial hypertension [16]. In addition, MSC-Exos suppress glycolysis in alveolar macrophages, thereby mitigating lipopolysaccharide-induced acute respiratory distress syndrome [17]. Whether MSC-Exos relieve renal fibrosis through the mitigation of glycolysis and the potential mechanisms involved remain elusive.

In the present study, we demonstrated that MSC-Exos alleviated renal fibrosis by attenuating glycolysis in TECs. Mechanistically, we found that the expression of miR-21a-5p was significantly elevated after MSC-Exos treatment and that PFKM acted as a downstream target of miR-21a-5p in the regulation of glycolysis. Altogether, our results demonstrated a correlation among MSC-Exos, glycolysis and renal fibrosis, and we further explored the potential molecular mechanisms. Our findings revealed a novel role of MSC-Exos in inhibiting glycolysis, thereby providing new insight into renal fibrosis therapy.

## Results

### Isolation and identification of MSC-Exos

After being cultured as described in the Materials and Methods, bone marrow MSCs were identified by morphology, multilineage differentiation abilities and surface markers. MSCs showed a typical spindle-like morphology by optical microscopy (Fig. S1A). The adipogenic and osteogenic differentiation of MSCs was identified by Oil Red O and Alizarin Red staining, respectively (Fig. S1B). Flow cytometry revealed that MSCs were positive for CD29 and CD44 but negative for CD31 and CD117 (Fig. S1C).

**Fig. 1:**
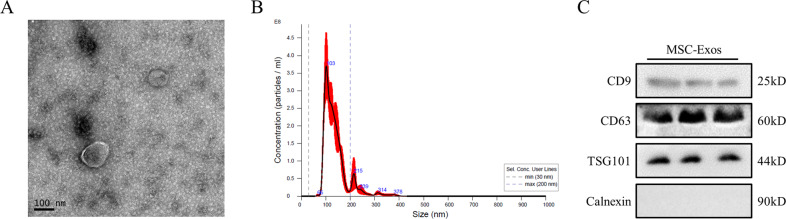
Isolation and identification of MSC-Exos. **A** The morphology of MSC-Exos was observed by TEM (scale bar=100 nm). Representative images from one experiment out of three are shown. **B** Representative results of NTA of MSC-Exos from three independent experiments. **C** Western blot analysis of exosomal protein markers (CD9, CD63 and TSG101) and an endoplasmic reticulum marker (calnexin) in MSC-Exos.

Exosomes were characterized by Transmission electron microscopy (TEM), nanoparticle tracking analysis (NTA) and western blot analysis. TEM showed that MSC-Exos displayed a round-shaped morphology of approximately 100 nm with a typical bilayer membrane structure (Fig. 1A and S2A). The peak diameter of the particles was 103 nm, and the median diameter was 137.2 nm as assessed by NTA (Fig. 1B and S2B). Western blot analysis indicated that MSC-Exos expressed exosome-related proteins, such as CD9, CD63, and TSG101, while calnexin, an endoplasmic reticulum marker, was absent in MSC-Exos (Fig. 1C and S5A).

### MSC-Exos alleviate UUO-induced renal fibrosis in mice

**Fig. 2:**
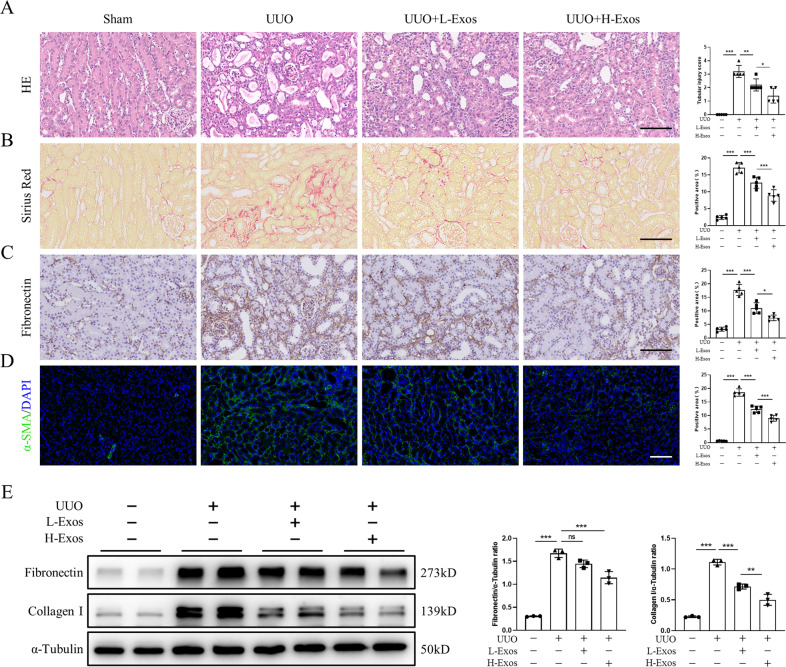
MSC-Exos alleviate UUO-induced renal fibrosis in mice. Mice were injected with MSC-Exos (50 µg or 100 µg) or PBS on Day 0 after UUO induction. Renal tissues from sham, UUO, L-Exos or H-Exo-treated mice were sampled on Day 7 after UUO induction. **A** Tubular injury was assessed by H&E staining (*n* = 5 mice per group; scale bar=100 μm). **B** Deposition of collagenous fibers in the renal interstitium was evaluated by Sirius Red staining (*n* = 5 mice per group; scale bar=100 μm). **C** Immunohistochemistry staining of fibronectin in kidney tissues (*n* = 5 mice per group; scale bar=100 μm). **D** Immunohistochemistry staining of α-SMA in kidney tissues (*n* = 5 mice per group; scale bar=100 μm). Representative images from one experiment out of three are shown. **E** Protein levels of fibronectin and collagen I in kidney tissues were assessed by western blot analysis (*n* = 3 mice per group). Data are presented as the mean ± SD from three independent experiments. ^*^p < 0.05, ^**^p < 0.01 and ^***^p < 0.001.

To investigate the therapeutic effects of MSC-Exos on renal fibrosis, we established a mouse model of renal fibrosis induced by UUO. Mice were administered low-dose MSC-Exos (L-Exos; 50 μg), high-dose MSC-Exos (H-Exos; 100 μg), or phosphate-buffered saline (PBS) via tail vein injection immediately after resuscitation. Compared to the sham group, UUO resulted in atrophy of TECs, loss of brush borders and significant dilatation of the tubular lumen, and these UUO-induced effects were ameliorated by MSC-Exos in a dose-dependent manner (Fig. 2A). Sirius red and immunohistochemistry staining showed that MSC-Exos markedly reduced the deposition of collagenous fibers and extracellular matrix (ECM) in the renal interstitium (Fig. 2B, C).

Because aberrant proliferation of myofibroblasts is a typical characteristic of renal fibrosis, we examined alpha smooth muscle actin (α-SMA), a marker of myofibroblasts, to assess the effect of MSC-Exos on the activation of myofibroblasts. Immunofluorescence staining indicated that the expression of α-SMA was reduced after MSC-Exos treatment, which indicated that MSC-Exos inhibited the activation of myofibroblasts (Fig. 2D). Western blot analysis revealed that the levels of ECM proteins, such as fibronectin and collagen I, were decreased after treatment with MSC-Exos (Fig. 2E and S5B). These results suggested that MSC-Exos alleviate UUO-induced murine renal fibrosis.

### MSC-Exos attenuate glycolysis in tubular epithelial cells

**Fig. 3:**
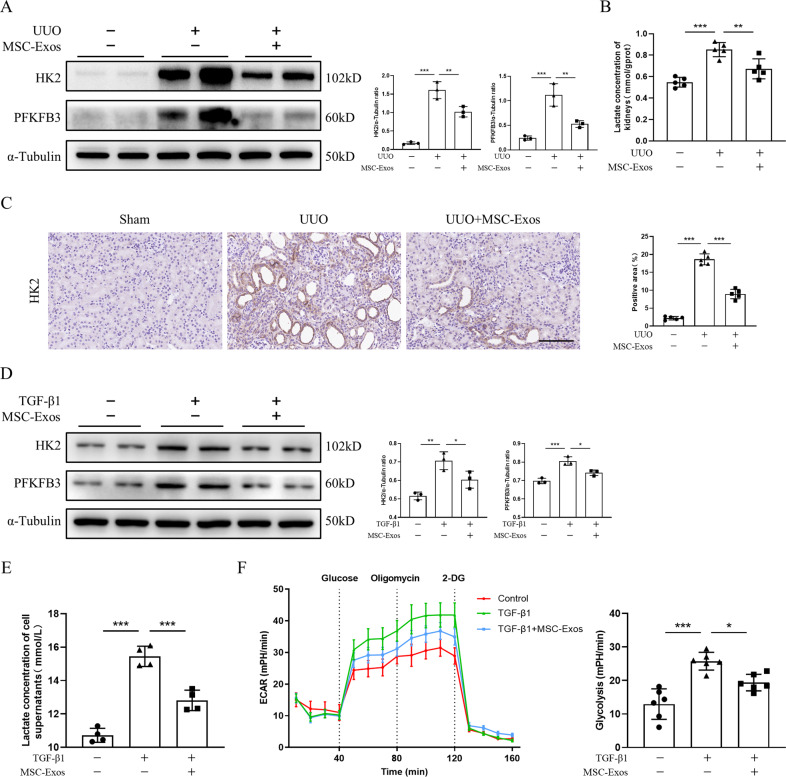
MSC-Exos attenuate glycolysis in tubular epithelial cells. MSC-Exos (100 µg) or PBS was administered intravenously on Day 0 after UUO induction. Kidneys were isolated from sham, UUO and MSC-Exo-treated mice on Day 7. TCMK-1 cells were incubated with TGF-β1 with or without MSC-Exos (20 µg). Cells and cell supernatants were collected at 48 h. **A** The expression of HK2 and PFKFB3 in renal tissues was measured by western blot analysis (*n* = 3 mice per group). **B** The lactate concentration of kidney tissue homogenates was quantified by a lactate assay kit (*n* = 5 mice per group). **C** Immunohistochemistry staining of HK2 in kidney tissues (*n* = 5 mice per group; scale bar=100 μm). Representative images from one experiment out of three are shown. **D** Protein levels of HK2 and PFKFB3 in TCMK-1 cells were assessed by western blot analysis (*n* = 3). **E** Lactate concentration in the supernatant of TCMK-1 cells was assayed by a lactate assay kit (*n* = 4). **F** Real-time ECAR of PBS-, TGF-β1-, and MSC-Exo-treated TCMK-1 cells was determined by a Seahorse XF96 Extracellular Flux Analyzer (*n* = 6). Data are presented as the mean ± SD from three independent experiments. ^*^p < 0.05, ^**^p < 0.01 and ^***^p < 0.001.

Metabolic reprogramming to aerobic glycolysis is a feature of renal fibrosis. Therefore, we investigated whether MSC-Exos interfere with glycolysis in renal fibrosis. MSC-Exos (100 μg) were administered intravenously to test this hypothesis. As expected, MSC-Exos markedly downregulated the expression of glycolysis-related enzymes, such as hexokinase 2 (HK2) and 6-phosphofructo-2-kinase/fructose-2,6-bisphosphatase 1 (PFKFB1) (Fig. S3A). Furthermore, the decreased levels of HK2 and PFKFB3 induced by MSC-Exos were confirmed by western blot analysis (Fig. 3A and S5C). Because lactate is the final metabolite of the glycolytic process, we assayed the concentration of lactate in renal tissues. The lactate level of fibrotic kidneys was significantly reduced after MSC-Exo treatment (Fig. 3B). Subsequently, immunohistochemistry staining was performed to identify the site where glycolysis occurs in the kidney, which revealed that HK2 was predominantly expressed in TECs, suggesting that a metabolic switch to glycolysis occurs in TECs during renal fibrosis (Fig. 3C).

Given that glycolysis takes place mainly in TECs in the process of renal fibrosis, we investigated whether MSC-Exos alleviate glycolysis in TECs ex vivo. We established a glycolysis model in the mouse TEC line, TCMK-1, induced by transforming growth factor-β1 (TGF-β1) as previous studies have demonstrated that TGF-β1 induces glycolysis in TECs [18, 19]. As expected, TGF-β1 significantly increased the expression of HK2 and PFKFB1 in TCMK-1 cells, while MSC-Exos diminished their levels (Fig. S3B). Western blot analysis revealed that the levels of HK2 and PFKFB3 were reduced after treatment with MSC-Exos (Fig. 3D and S5D). Similarly, the lactate concentration of the cell supernatants was decreased after treatment with MSC-Exos (Fig. 3E). To further investigate the effect of MSC-Exos on glycolysis, we assessed the extracellular acidification rate (ECAR) of TCMK-1 cells with a Seahorse Extracellular Flux Analyzer as ECAR indirectly reflects the glycolytic capacity of cells. The results showed that the ECAR of TCMK-1 cells induced by TGF-β1 was markedly elevated, which was significantly diminished by MSC-Exos (Fig. 3F). Collectively, these findings suggested that MSC-Exos alleviate glycolysis in TECs both in vivo and in vitro.

### MSC-Exos attenuate glycolysis in tubular epithelial cells by inhibiting PFKM through miR-21a-5p

**Fig. 4:**
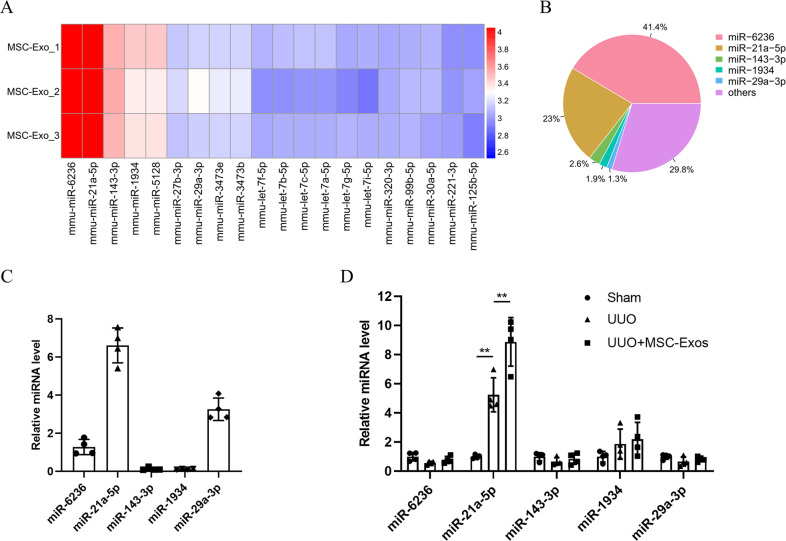
miR-21a-5p is enriched in MSC-Exos. **A** Exosomal miRNA sequencing revealed the top 20 most abundant miRNAs in MSC-Exos. **B** Relative percentages of different miRNAs in total miRNA reads. **C** qPCR analysis of the top 5 miRNAs in MSC-Exos (*n* = 4). **D** Relative expression of the top 5 miRNAs in MSC-Exos in kidney tissues from sham, UUO and MSC-Exo-treated mice (*n* = 4 mice per group). Data are presented as the mean ± SD from three independent experiments. ^**^p < 0.01.

Because exosomes regulate many physiological activities through miRNAs, we investigated whether MSC-Exos regulate glycolysis via miRNAs by analyzing the exosomal miRNA expression profiles of MSC-Exos by miRNA sequencing. The top five most abundant miRNAs in MSC-Exos were miR-6236, miR-21a-5p, miR-143-3p, miR-1934 and miR-29a-3p, accounting for approximately 70% of the total miRNA reads (Fig. 4A, B). We next detected the expression of the top five miRNAs in MSC-Exos. miR-21a-5p was highly expressed in MSC-Exos as determined by qPCR (Fig. 4C). To investigate whether miRNAs were involved in the renoprotective effects of MSC-Exos and which miRNAs played a role, we further measured the expression of these miRNAs in MSC-Exo-treated kidney tissues. MSC-Compared to the sham and UUO groups, Exos significantly upregulated the expression of miR-21a-5p (Fig. 4D).

**Fig. 5:**
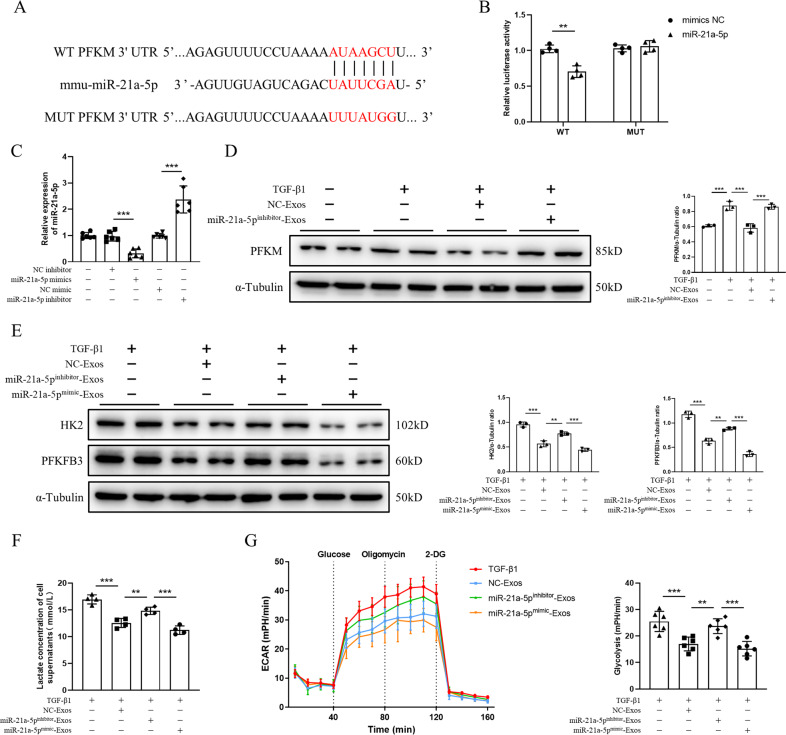
MSC-Exos attenuate glycolysis in tubular epithelial cells by inhibiting PFKM through miR-21a-5p. **A** The binding sites of miR-21a-5p and PFKM as well as the sequence of the MUT PFKM 3’UTR. **B** Luciferase activity of WT or MUT PFKM reporter was assessed 48 h after transfection (*n* = 4). **C** Relative expression of miR-21a-5p in MSCs transfected with miR-21a-5p inhibitor, NC inhibitor, miR-21a-5p mimic, or NC mimic by qPCR (*n* = 6). **D** The expression of PFKM was assessed by western blot analysis in PBS-, TGF-β1-, NC-Exo- and miR-21a-5p^inhibitor^-Exo-treated TCMK-1 cells (*n* = 3). **E** The expression of HK2 and PFKFB3 in TCMK-1 cells was assessed by western blot analysis (*n* = 3). **F** Lactate concentration in the supernatant of TCMK-1 cells (*n* = 4). **G** ECAR of TGF-β1-, NC-Exo-, miR-21a-5p^inhibitor^-Exo- and miR-21a-5p^mimic^-Exo-treated TCMK-1 cells (*n* = 6). Data are presented as the mean ± SD from three independent experiments. ^**^p < 0.01 and ^***^p < 0.001.

To explore the role of miR-21a-5p, we predicted the potential target genes of miR-21a-5p that may be involved in the glycolytic process through the miRDB and TargetScan databases. Both databases suggested that PFKM is a potential target gene of miR-21a-5p (Fig. 5A). To confirm the association between miR-21a-5p and PFKM, we conducted a luciferase reporter assay. Overexpression of miR-21a-5p significantly decreased the activity of the wild-type (WT) PFKM 3’ untranslated region (UTR) luciferase reporter. However, the activity of the mutant (MUT) PFKM 3’UTR luciferase reporter was not significantly altered (Fig. 5B), indicating that PFKM is a target gene of miR-21a-5p.

To investigate the role of miR-21a-5p in MSC-Exos, MSCs were transfected with a miR-21a-5p inhibitor and miR-21a-5p mimic. Compared to the negative control (NC) inhibitor or control group, transfection with the miR-21a-5p inhibitor significantly reduced the level of miR-21a-5p, while transfection of miR-21a-5p mimic significantly upregulated the expression of miR-21a-5p (Fig. 5C). Exosomes of miR-21a-5p mimic-transfected MSCs (miR-21a-5p^mimic^-Exos) and miR-21a-5p inhibitor-transfected MSCs (miR-21a-5p^inhibitor^-Exos) were subsequently isolated from cell supernatants. NC-Exos significantly decreased the expression of PFKM in TCMK-1 cells treated with TGF-β1, which was reversed by treatment with miR-21a-5p^inhibitor^-Exos (Fig. 5D, S4, and S5E). The expression of other glycolysis-related enzymes was also significantly upregulated by miR-21a-5p^inhibitor^-Exos, while miR-21a-5p^mimic^-Exos further inhibited glycolysis in TCMK-1 cells compared to the NC-Exo group (Fig. 5E and S5F). Moreover, treatment with miR-21a-5p^inhibitor^-Exos increased the lactate concentration of TCMK-1-cell supernatants compared to NC-Exos, whereas miR-21a-5p^mimic^-Exos eliminated this effect (Fig. 5F). Consistently, the ECAR of the TCMK-1 cells was significantly increased in the miR-21a-5p^inhibitor^-Exos group compared to the NC-Exos and miR-21a-5p^mimic^-Exos group (Fig. 5G). Taken together, these results indicated that MSC-Exos attenuate glycolysis in TECs by inhibiting PFKM through miR-21a-5p.

### miR-21a-5p is essential for the renoprotective effects of MSC-Exos

**Fig. 6:**
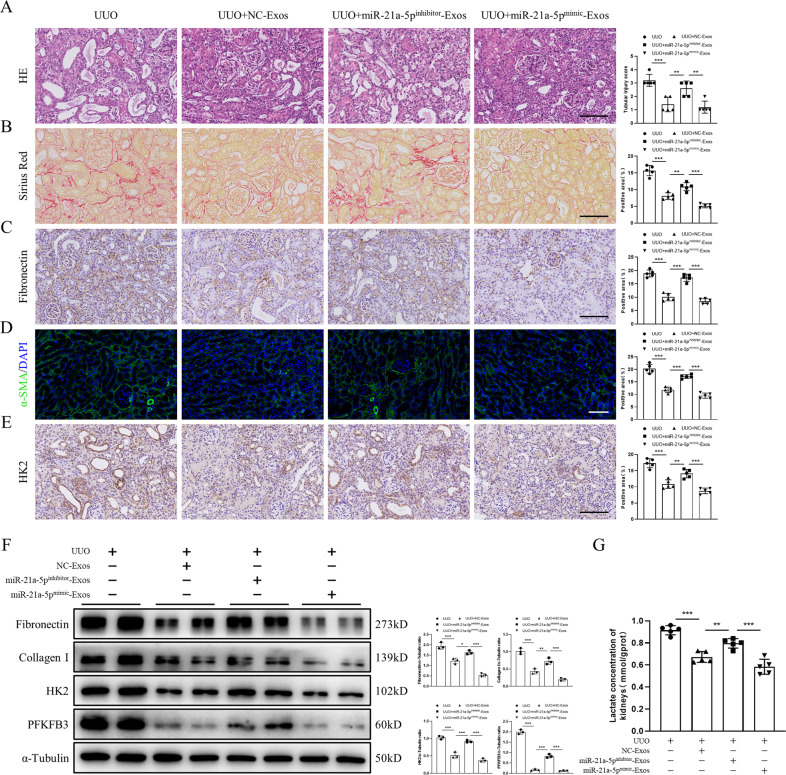
miR-21a-5p is essential for the renoprotective effects of MSC-Exos. Mice were injected with NC-Exos, miR-21a-5p^inhibitor^-Exos, miR-21a-5p^mimic^-Exos or PBS on Day 0 after UUO induction. Renal tissues were harvested on Day 7 after UUO. **A** Tubular injury was assessed by H&E staining (*n* = 5 mice per group; scale bar=100 μm). **B** Deposition of collagenous fibers in the renal interstitium was evaluated by Sirius Red staining (*n* = 5 mice per group; scale bar=100 μm). **C** Immunohistochemistry staining of fibronectin in kidney tissues (*n* = 5 mice per group; scale bar=100 μm). **D** Immunohistochemistry staining of α-SMA in kidney tissues (*n* = 5 mice per group; scale bar=100 μm). **E** Immunohistochemistry staining of HK2 in kidney tissues (*n* = 5 mice per group; scale bar=100 μm). Representative images from one experiment out of three are shown. **F** The expression of fibronectin, collagen I, HK2 and PFKFB3 in kidney tissues was assessed by western blot analysis (*n* = 3 mice per group). **G** The lactate concentration of kidney tissue homogenates was quantified by a lactate assay kit (*n* = 5 mice per group). Data are presented as the mean ± SD from three independent experiments. ^*^p < 0.05, ^**^p < 0.01 and ^***^p < 0.001.

To further explore whether miR-21a-5p is involved in MSC-Exo-mediated renoprotective effects in vivo, mice were administered miR-21a-5p^inhibitor^-Exos or miR-21a-5p^mimic^-Exos. Compared to the NC-Exo group, mice treated with miR-21a-5p^inhibitor^-Exos showed renewed atrophy of TECs, loss of brush borders and dilation of the tubular lumen, while MSC-Exo-overexpressing miR-21a-5p significantly attenuated these pathological changes (Fig. 6A). Sirius red and immunohistochemistry staining revealed that knockdown of miR-21a-5p significantly increased the deposition of collagenous fibers and ECM, which was significantly counteracted by miR-21a-5p^mimic^-Exos (Fig. 6B, C). Immunofluorescence staining indicated that miR-21a-5p^inhibitor^-Exos reversed the inhibitory effect of miR-21a-5p^mimic^-Exos on α-SMA expression (Fig. 6D). Furthermore, knockdown of miR-21a-5p significantly enhanced the expression of HK2 in TECs compared to that in TECs treated with miR-21a-5p mimic (Fig. 6E). Similarly, downregulation of miR-21a-5p significantly upregulated the levels of ECM proteins and glycolysis-related enzymes (Fig. 6F and S5G). In addition, miR-21a-5p^inhibitor^-Exos reversed the decrease in lactate concentration induced by NC-Exos and miR-21a-5p^mimic^-Exos (Fig. 6G). These results suggested that miR-21a-5p is essential for the renoprotective and antiglycolytic effects of MSC-Exos.

## Discussion

**Fig. 7:**
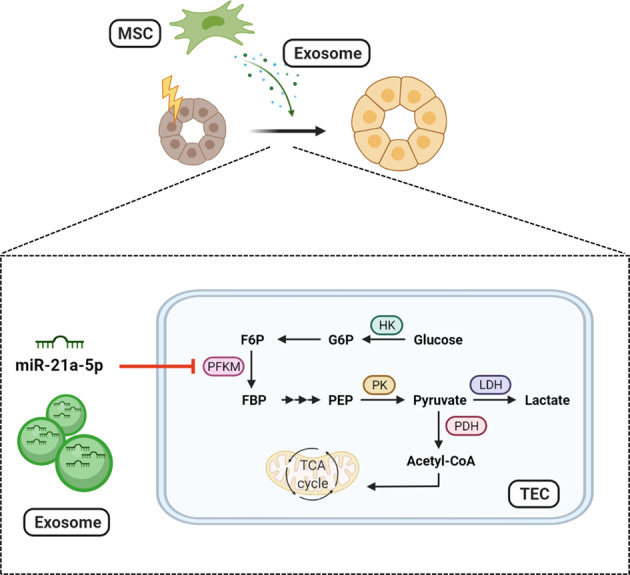
Schematic diagram of the effects of MSC-Exos on glycolysis and renal fibrosis. In the process of renal fibrosis, TECs undergo a metabolic shift to aerobic glycolysis, and MSC-Exos are able to attenuate renal fibrosis by inhibiting glycolysis in TECs via the miR-21a-5p/PFKM pathway. The schematic diagram was created by biorender.com.

In the present study, we found that MSC-Exos effectively alleviated UUO-induced renal fibrosis. Importantly, we demonstrated for the first time that MSC-Exos ameliorated renal fibrosis by attenuating glycolysis in TECs. Further studies showed that the antiglycolytic effects of MSC-Exos were achieved miR-21a-5p-mediated targeting of PFKM (Fig. 7).

Renal fibrosis is the common pathological outcome of almost all chronic kidney diseases and indicates a poor prognosis. Cytotherapy has emerged as a promising strategy for the treatment of renal fibrosis, in which MSCs have played an increasingly important role [20]. Accumulating evidence shows that MSCs exert pleiotropic effects, mainly in a paracrine manner [10, 21]. MSC-derived exosomes have emerged as a promising strategy for the treatment of renal fibrosis due to their antiapoptotic, anti-inflammatory and proangiogenic effects [22–24]. In the present study, we identified a novel role for MSC-Exos in renal fibrosis, in which MSC-Exos alleviate renal fibrosis by inhibiting glycolysis in TECs. Although previous studies have shown that MSC-Exos may inhibit glycolysis in other diseases [16, 17], this is the first report suggesting that MSC-Exos may exert an antiglycolytic effect in renal fibrosis.

TECs are highly metabolically active cells with reabsorption, water-electrolyte maintenance and acid-base balance functions. Healthy TECs rely on fatty acids to meet their energy demands. However, under stimuli, such as ischemia, obstruction and drugs, fatty acid oxidation malfunctions in TECs [25, 26]. Fatty acid oxidation dysfunction is generally accompanied by intracellular lipid deposition and a shift in cellular metabolism toward aerobic glycolysis. Cao et al. demonstrated that TECs utilizes glycolysis to provide energy during kidney fibrosis [18]. Although aerobic glycolysis provides energy and substrates for the proliferation of TECs, it also promotes the progression of renal fibrosis. Cai et al. found that a metabolic switch from fatty acid oxidation to glycolysis occurs in patients with diabetic nephropathy [27]. As expected, we found that the gene and protein levels of glycolysis-related enzymes were significantly upregulated in murine fibrotic kidneys, and we found a marked increase in lactate concentration, confirming a metabolic switch to aerobic glycolysis in renal fibrosis. In addition, we further found that this metabolic shift occurred mainly in TECs. TGF-β1, which is a potent profibrotic factor, a metabolic regulator and a potent activator of glycolysis, promotes glycolysis by directly inducing the expression of glycolytic enzymes, such as HK and PFK [28, 29]. Moreover, TGF-β1 inhibits the conversion of pyruvate to acetyl coenzyme A by stabilizing HIF-1α, which in turn activates PDK1, thereby indirectly promoting glycolysis [30]. Previous studies have shown that TGF-β1 induces glycolysis in TECs [18, 19]. Consistent with these studies, we found that TGF-β1 not only promoted the expression of HK2 and PFKFB3 but also significantly increased the ECAR and lactate concentration in TCMK-1 cells. More importantly, MSC-Exos alleviated glycolysis in TECs both in vivo and in vitro.

miRNAs are a class of endogenous noncoding single-stranded RNA molecules with a length of approximately 22 nucleotides that are widely found in eukaryotes, and they perform important roles in organism development, cell differentiation and homeostasis [31–33]. Recent studies have demonstrated that MSC-Exos exert protective effects through miRNAs in multiple kidney diseases [34–36]. Therefore, we speculated that certain miRNAs contained in exosomes may be involved in the alleviation of renal fibrosis. As expected, we found that miR-6236 and miR-21a-5p were enriched in MSC-Exos by miRNA sequencing. Further investigation found that miR-21a-5p but not miR-6236 was significantly elevated in the kidney after treatment with MSC-Exos. miR-21a-5p, which is located on chromosome 11 and has a length of 22 nucleotides, has been shown to play a positive regulatory role in a variety of diseases. Luther et al. validated that MSC-Exos attenuates myocardial ischemia–reperfusion injury by inhibiting the apoptosis of cardiomyocytes via miR-21a-5p [37]. Xin et al. demonstrated that EVs derived from MSCs regulate the polarization of microglia and macrophages by delivering miR-21a-5p, thereby mitigating hypoxic-ischemic injury [38]. In agreement with these studies, we found that knockdown of miR-21a-5p exacerbated glycolysis in TECs and renal fibrosis, while overexpression of miR-21a-5p significantly attenuated this effect, suggesting that miR-21a-5p is essential for renoprotection. Mechanistically, miR-21a-5p binds to the 3’UTR of PFKM to suppress its expression. PFKM is the second rate-limiting enzyme in glycolysis, catalyzing an irreversible reaction and serving an essential regulatory role in glycolysis [39, 40]. Taken together, the present findings revealed that MSC-Exos are involved in the regulation of glycolysis through the miR-21a-5p/PFKM signaling pathway, thereby ameliorating renal fibrosis.

The present study had several limitations. First, we focused on the regulatory role of MSC-Exos on glycolysis without further exploring the alterations of oxidative phosphorylation or fatty acid oxidation in the kidney. Second, we only investigated the regulatory role of miR-21a-5p on glycolysis in the present study, and other targets of miR-21a-5p or other miRNAs may also exert nephroprotective effects via other modalities. We speculate that miRNAs may function in different pathophysiological processes in addition to glycolysis to alleviate renal fibrosis. Further experiments are warranted to elaborate on these issues.

Taken together, the present results demonstrated that MSC-Exos ameliorates renal fibrosis by attenuating the glycolysis of TECs through miR-21a-5p-mediated targeting of PFKM. These findings identified a novel mechanism of MSC-Exos in renal repair, which may provide a promising therapeutic strategy for renal fibrosis.

## Materials and methods

### Culture and identification of mesenchymal stem cells

Mouse bone marrow MSCs were purchased from Cyagen Biosciences (Suzhou, China) and were routinely cultured in DMEM (Gibco, Carlsbad, CA, USA) supplemented with 10% fetal bovine serum (FBS; HyClone, Logan, UT, USA) and 1% penicillin-streptomycin (Gibco). The morphology of the cells was observed by optical microscopy. Flow cytometry was used to identify cell phenotypes with an MSC surface marker assay kit (Cyagen Biosciences). To verify the osteogenic differentiation and adipogenic differentiation ability, an MSC osteogenic differentiation kit and adipogenic differentiation kit (both from Cyagen Biosciences) were used according to the manufacturer’s instructions.

### Isolation and identification of MSC-Exos

MSCs were cultured in DMEM with 10% exosome-depleted FBS (SBI, Palo Alto, CA, USA) for 48 h, and the supernatants were subsequently collected. Exosomes were isolated by ultracentrifugation. In brief, the supernatants were centrifuged at 300 × *g* for 10 min, 2000 × *g* for 10 min, 10000 × *g* for 30 min and 100,000 × *g* for 70 min. The pellets were then resuspended in PBS (Gibco) and stored at −80 °C.

The morphology of the exosomes was observed by TEM (FEI, Hillsboro, OR, USA). NTA (Particle Metrix, Inning am Ammersee, Germany) was used to assess the size and concentration of the exosomes. Common markers of exosomes (CD9, CD63 and TSG101) and an endoplasmic reticulum marker (calnexin) were assessed by western blot analysis.

### Mouse models and treatments

Male C57BL/6 mice (6 weeks old; 20–25 g) were purchased from Shanghai Jiesijie Laboratory Animal Co., Ltd. (Shanghai, China) and maintained in a specific pathogen-free-grade animal room. Mice were divided randomly into 4 different groups (*n* = 5): 1) Sham group; 2) UUO group; 3) UUO + L-Exos group; 4) UUO + H-Exos group. The UUO model was established as previously described. Briefly, a midline incision was performed to fully expose the left kidney. The left ureter was carefully separated, ligated and then dissected between the ligatures. In sham mice, the left kidney was also exposed but the ureter was not ligated. Mice were administered PBS, L-Exos or H-Exos via tail vein injection immediately after resuscitation. To investigate the role of miR-21a-5p in MSC-Exos, mice were injected intravenously with PBS, NC-Exos (100 μg), miR-21a-5p^inhibitor^-Exos, or miR-21a-5p^mimic^-Exos (100 μg).

### Cell culture and treatments

The mouse TEC line, TCMK-1, was purchased from FuHeng Biology (Shanghai, China) and cultured in DMEM/F12 (Gibco) supplemented with 10% FBS (HyClone). TCMK-1 cells were incubated with TGF-β1 (PeproTech, Rocky Hill, NJ, USA) at a concentration of 10 ng/ml for 48 h to establish a glycolysis model in vitro. TCMK-1 cells were then treated with MSC-Exos (20 μg) to investigate their effects on glycolysis.

### RNA extraction and quantitative real-time PCR

Total RNA was extracted from cells or kidney tissues using TRI reagent (Sigma–Aldrich, St. Louis, MO, USA). To extract miRNA from exosomes, the miRNeasy Serum/Plasma Kit (QIAGEN, Redwood City, CA, USA) was used. cDNA was synthesized using a Reverse Transcription Kit (Yeasen Biotechnology, Shanghai, China) or a Stem–Loop miRNA cDNA Synthesis Kit (Vazyme, Nanjing, China). Quantitative real-time PCR (qPCR) was performed using a SYBR Green Master Mix (Yeasen Biotechnology) or a miRNA qPCR kit (Vazyme) in a QuantStudio 5 instrument (Applied Biosystems, Foster City, CA, USA). GAPDH or U6 was used as the endogenous control. The primer sequences are shown in Table S1.

### Western blot analysis

Equal amounts of protein extracted from cells and kidney tissues were subjected to electrophoresis and then transferred to a polyvinylidene fluoride membrane. The primary antibodies against CD9, CD63, TSG101, fibronectin, collagen I, HK2, PFKFB3, PFKM and α-tubulin were obtained from Abcam (Cambridge, UK). The primary antibody against calnexin was purchased from Cell Signaling Technology (Danvers, MA, USA).

### Histologic analysis

Renal tissues were fixed in 10% formalin, embedded in paraffin and sliced into 5-μm-thick sections. After deparaffinization and rehydration, sections were stained with hematoxylin and eosin (H&E) and Sirius red. The area at the junction of the cortex and medulla in the section was selected for histological analysis. H&E staining was used to assess tubular injury using the following criteria: 0, no damage; 1, <25% damage; 2, 25–50% damage; 3, 50–75% damage; and 4, >75% damage. Sirius red staining was performed to evaluate the deposition of collagenous fibers in the renal interstitium. The positive area (red) of Sirius red staining was semiquantified using ImageJ. All histologic analysis were performed by two independent researchers who were blinded to the groupings.

### Immunohistochemistry staining

For immunohistochemistry staining, sections were deparaffinized, rehydrated and subjected to antigen retrieval. After inactivation of endogenous peroxidase by 3% hydrogen peroxide, sections were blocked with 10% normal goat serum for 10 min followed by incubation with primary antibodies against fibronectin and HK2 (both from Abcam) at 4 °C overnight. After incubation with biotin-labeled secondary antibody and streptavidin-peroxidase complex (both from Solarbio, Beijing, China), the nuclei were counterstained with hematoxylin (Solarbio). ImageJ was used for semiquantitative analysis of the positive area (brown) of immunohistochemical staining.

### Immunofluorescence staining

After deparaffinization, rehydration, and blocking with goat serum, kidney sections were incubated with an anti-α-SMA antibody (Abcam) at 4 °C overnight, followed by incubation with Alexa Fluor^®^ 488-conjugated goat anti-rabbit IgG (Abcam). DAPI (Sigma–Aldrich) was used to visualize the nuclei. The area of α-SMA^+^ was quantified by ImageJ.

### Glycolysis analysis

A Seahorse XF96 Extracellular Flux Analyzer (Agilent Technologies, Santa Clara, CA, USA) was used for real-time analysis of ECAR. Seahorse XF calibrant (Agilent Technologies) was added to the utility plate, and the sensor cartridge and utility plate were placed in a non-CO_2_ incubator overnight. TCMK-1 cells were seeded in a Seahorse XF96 culture microplate (Agilent Technologies) at a density of 10^5^ cells per well overnight. The microplate was replaced with assay media and placed in a CO_2_-free incubator for 1 h followed by the addition of glucose, oligomycin and 2-DG into the injection ports of the sensor cartridge.

### Lactate assay

The lactate concentration of the cell supernatant and kidney tissues was measured using a lactate assay kit (Nanjing Jiancheng Bioengineering Institute, Nanjing, China) according to the manufacturer’s instructions.

### Exosomal miRNA sequencing

Library construction and sequencing of exosomal miRNA were performed by Obio Technology Corp., Ltd. (Shanghai, China). miRNAs were extracted with a TruSeq Small RNA Sample Prep Kit (Illumina, San Diego, CA, USA) and sequenced using HiSeq2000/2500 (Illumina).

### Cell transfection

Bone marrow MSCs were transfected with a miR-21a-5p inhibitor, NC inhibitor, miR-21a-5p mimic, or NC mimic at a concentration of 150 nM using Lipofectamine 3000 (Invitrogen, Carlsbad, CA, USA) according to the manufacturer’s instructions. After 48 h of transfection, qPCR was performed to assess the expression of miR-21a-5p. The miR-21a-5p inhibitor, NC inhibitor, miR-21a-5p mimic, and NC mimic were synthesized by GenePharma (Shanghai, China) using the following sequences: miR-21a-5p inhibitor, 5′-UCAACAUCAGUCUGAUAAGCUA-3′; and NC inhibitor, 5′-CAGUACUUUUGUGUAGUACAA-3′; miR-21a-5p mimic: sense, 5′-UAGCUUAUCAGACUGAUGUUG-3′ and antisense, 5′-AACAUCAGUCUGAUAAGCUAUU-3′; NC mimic: sense, 5′-UUCUCCGAACGUGUCACGUTT-3′ and antisense, 5′-ACGUGACACGUUCGGAGAATT-3′.

### Luciferase reporter assay

293 T cells were cotransfected with WT or MUT PFKM 3′ UTR luciferase vector and with miR-21a-5p mimic or NC mimic using GP-transfect-Mate (GenePharma). At 48 h after transfection, luciferase activity was assessed using a Dual-Luciferase Reporter Assay System kit (GenePharma).

### Statistical analysis

Statistical analysis was performed using GraphPad Prism version 8.3.0 (GraphPad Software, San Diego, CA, USA). Data are shown as the mean ± standard deviation (SD). The unpaired Student’s *t* test was used for comparisons between two groups, and one-way ANOVA was used for comparisons among three or more groups. *p* < 0.05 was considered significant.

## Supplementary information

Figure S1

Figure S2

Figure S3

Figure S4

Figure S5

Table S1

aj-checklist

Supplementary Figure Legends

## Data Availability

All data supporting this research has been included in this manuscript. Further inquiries can be directed to the corresponding author.
