# Supplementary figures and images for: Bone marrow mesenchymal stem cell-derived exosomal miR-21a-5p alleviates renal fibrosis by attenuating glycolysis by targeting PFKM
Source: Cell Death Dis. 2022 Oct 17;13(10):876. doi: 10.1038/s41419-022-05305-7 (PMC9576726; doi:10.1038/s41419-022-05305-7)

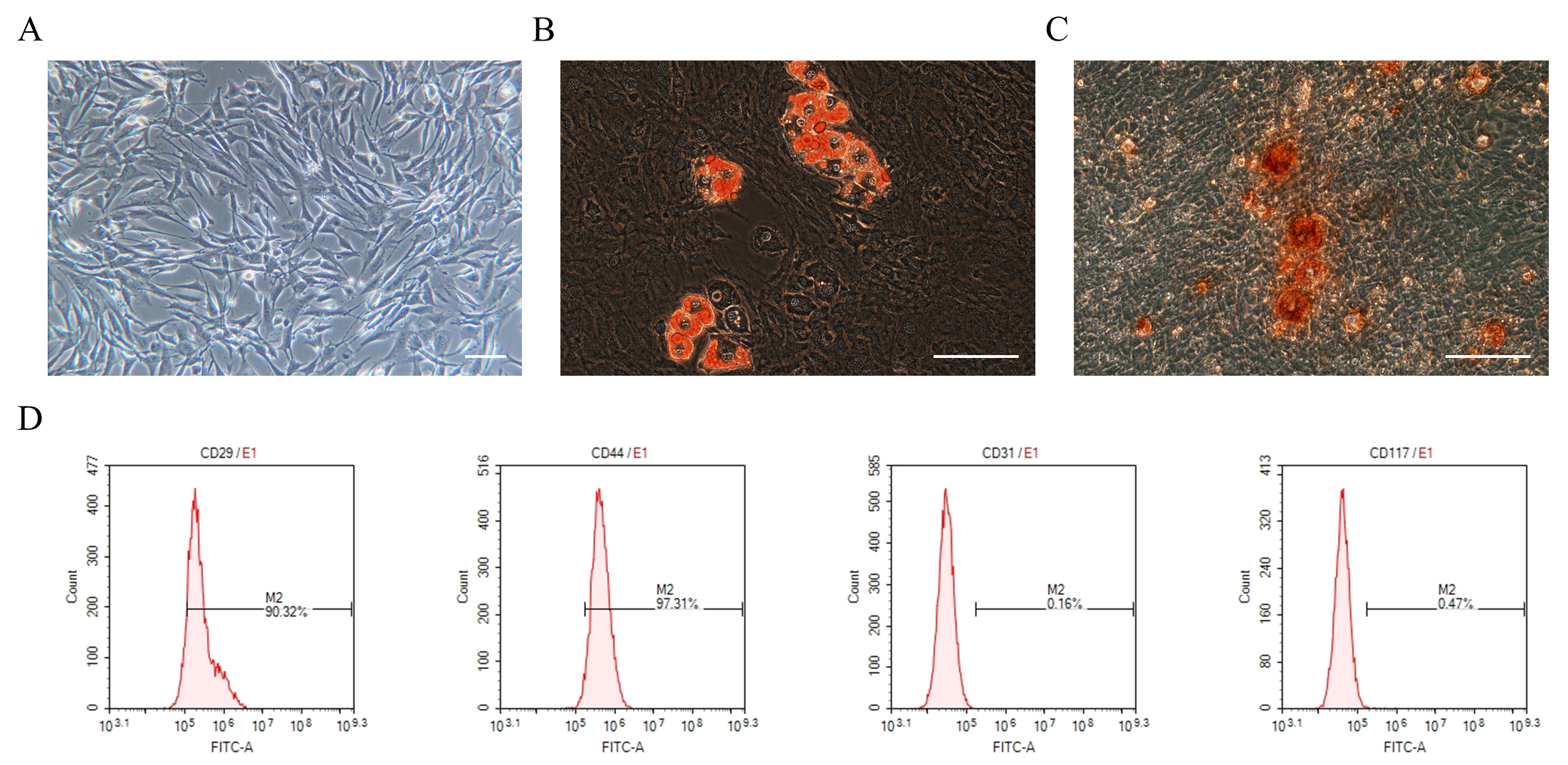

Supplement: Supplementary file 1 — Figure S1 [file 41419_2022_5305_MOESM1_ESM.tif]

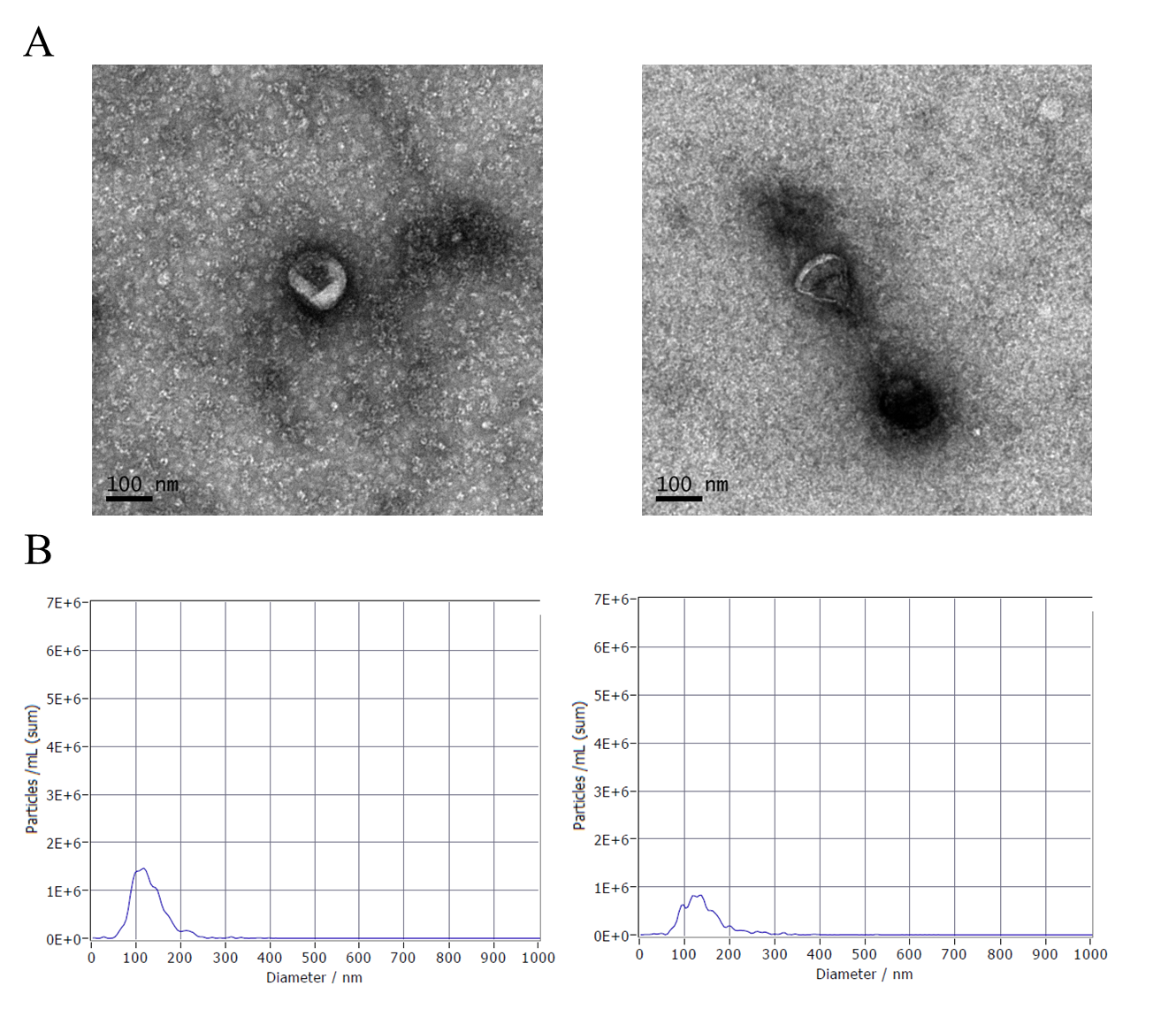

Supplement: Supplementary file 2 — Figure S2 [file 41419_2022_5305_MOESM2_ESM.tif]

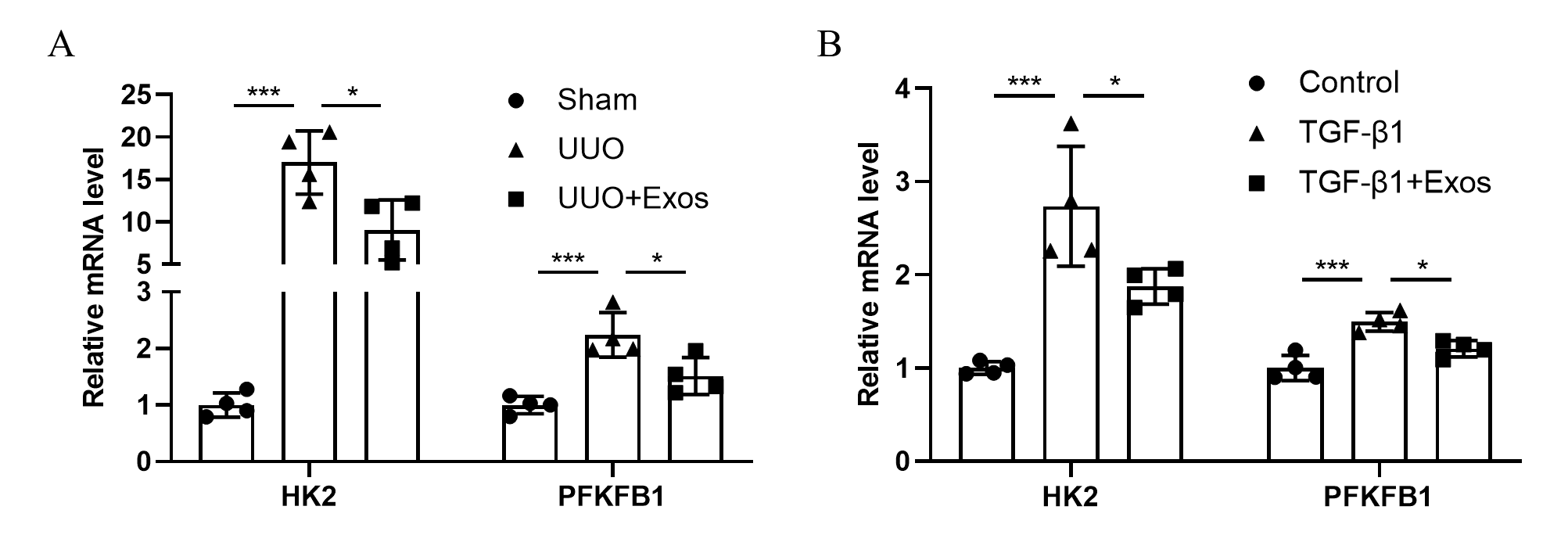

Supplement: Supplementary file 3 — Figure S3 [file 41419_2022_5305_MOESM3_ESM.tif]

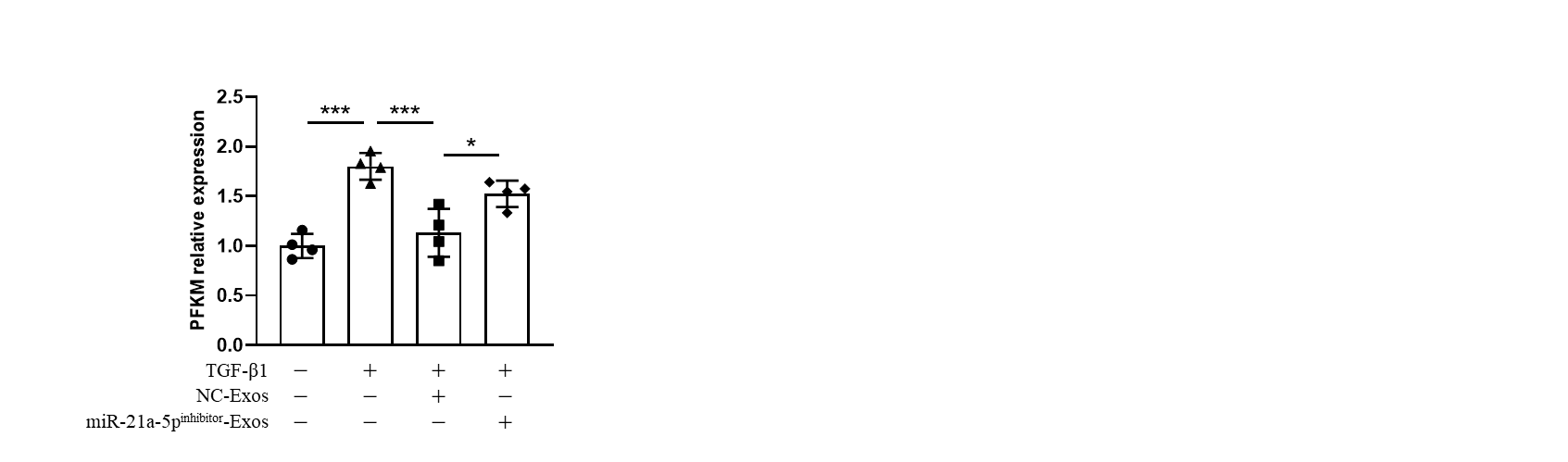

Supplement: Supplementary file 4 — Figure S4 [file 41419_2022_5305_MOESM4_ESM.tif]

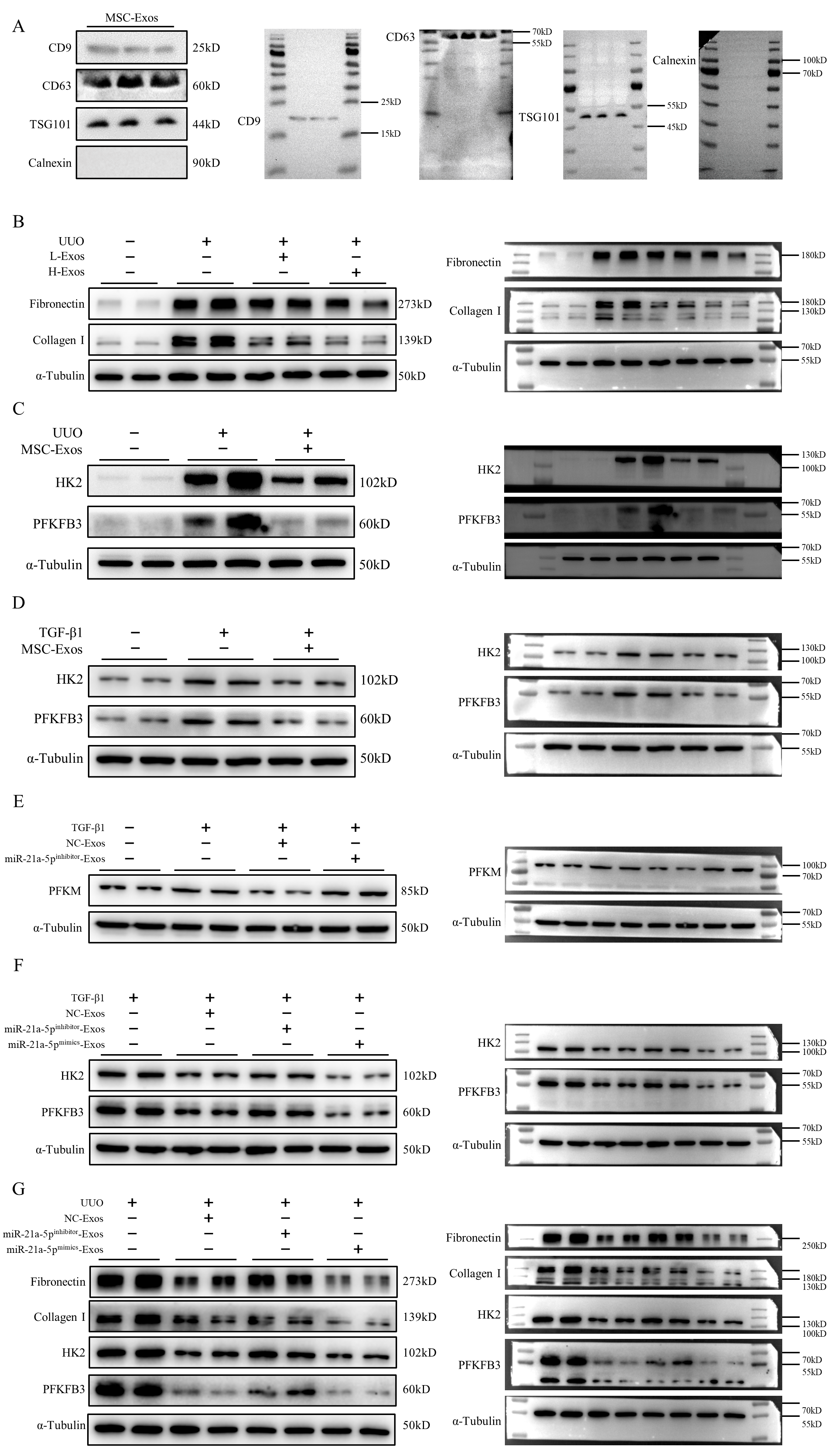

Supplement: Supplementary file 5 — Figure S5 [file 41419_2022_5305_MOESM5_ESM.tif]
