# Supplementary material for: Bone marrow mesenchymal stem cell-derived exosomal miR-21a-5p alleviates renal fibrosis by attenuating glycolysis by targeting PFKM
Source: Cell Death Dis. 2022 Oct 17;13(10):876. doi: 10.1038/s41419-022-05305-7 (PMC9576726; doi:10.1038/s41419-022-05305-7)
Supplement: Supplementary file 6 — Table S1 [file 41419_2022_5305_MOESM6_ESM.docx]

**Table S1. Primer sequences used in this study.**

| Target | Primer sequence (5'→3') |
| --- | --- |
| GAPDH | F: TCACCATCTTCCAGGAGCGAGAC |
|  | R: TGAGCCCTTCCACAATGCCAAAG |
| PFKFB1 | F: AGCCGTACAGCCTACTACCTCATG |
|  | R: TCTTCACAGACACCCGCATCAATC |
| HK2 | F: GTTTCACCTTCTCCTTCCCTTGCC |
|  | R: CCTCCTCTCCGTCCACCAGTTC |
| PFKM | F: ATGAATGCCGCTGTTCGCTCTAC |
|  | R: TCAAACTGCTTCCTGCCTTCCATC |
| U6 | F: CAGCACATATACTAAAATTGGAACG |
|  | R: ACGAATTTGCGTGTCATCC |
