# Supplementary material for: Bone marrow mesenchymal stem cell-derived exosomal miR-21a-5p alleviates renal fibrosis by attenuating glycolysis by targeting PFKM
Source: Cell Death Dis. 2022 Oct 17;13(10):876. doi: 10.1038/s41419-022-05305-7 (PMC9576726; doi:10.1038/s41419-022-05305-7)
Supplement: Supplementary file 8 — Supplementary Figure Legends [file 41419_2022_5305_MOESM8_ESM.docx]

**Supplementary** **Information**

**Figure S1. Identification of MSCs.** (A) The morphology of the MSCs was observed with an optical microscope (scale bar = 100 μm). (B) Adipogenic differentiation of MSCs was identified by Oil Red O staining (scale bar = 100 μm). (C) Osteogenic differentiation of MSCs was determined by Alizarin Red staining (scale bar = 100 μm). (D) Surface markers of MSCs (CD29, CD31, CD44, CD117 and Sca-1) were analyzed by flow cytometry.

**Figure S2. Identification of MSC-Exos.** (A) The morphology of MSC-Exos was observed by TEM (scale bar = 100 nm). (B) The results of NTA of MSC-Exos.

**Figure S3**. **MSC-Exos attenuate glycolysis both *in vivo* and *in vitro*.** (A) mRNA levels of HK2 and PFKFB1 in kidney tissues were assessed by qPCR (n = 4 mice per group). (B) Relative expression of HK2 and PFKFB1 in TCMK-1 cells was measured by qPCR (n = 4). Data are presented as the mean ± SD from three independent experiments. ***p* < 0.01 and ****p* < 0.001.

**Figure S4. MSC-Exos inhibit PFKM in tubular epithelial cells through miR-21a-5p.** The expression of PFKM was assessed by qPCR in PBS-, TGF-β1-, NC-Exo- and miR-21a-5p^inhibitor^-Exo-treated TCMK-1 cells (n = 4). Data are presented as the mean ± SD from three independent experiments. ***p* < 0.01 and ****p* < 0.001.

**Figure S5. Original full-length western blots.**
